# Improved YOLOv4 for Pedestrian Detection and Counting in UAV Images

**DOI:** 10.1155/2022/6106853

**Published:** 2022-07-14

**Authors:** Hao Kong, Zhi Chen, Wenjing Yue, Kang Ni

**Affiliations:** ^1^School of Computer, Nanjing University of Posts and Telecommunications, Nanjing, Jiangsu 210023, China; ^2^School of Telecommunications and Information Engineering, Nanjing University of Posts and Telecommunications, Nanjing, Jiangsu 210023, China

## Abstract

UAV (unmanned aerial vehicle) captured images have small pedestrian targets and loss of key information after multiple down sampling, which are difficult to overcome by existing methods. We propose an improved YOLOv4 model for pedestrian detection and counting in UAV images, named YOLO-CC. We used the lightweight YOLOv4 for pedestrian detection, which replaces the backbone with CSPDarknet-34, and two feature layers are fused by FPN (Feature Pyramid Networks). We expanded the perception field using multiscale convolution based on the high-level feature map and generated the population density map by feature dimension reduction. By embedding the density map generation method into the network for end-to-end training, our model can effectively improve the accuracy of detection and counting and make feature extraction more focused on small targets. Our experiments demonstrate that YOLO-CC achieves 21.76 points AP_50_ higher than that of the original YOLOv4 on the VisDrone2021-counting data set while running faster than the original YOLOv4.

## 1. Introduction

UAV remote sensing is widely used in agricultural and forestry plant protection, production monitoring, geographic mapping, public security inspection, emergency rescue, and other civil fields. With the continuous improvement of hardware performance, the application research of UAVs based on computer vision has attracted the attention of relevant experts and scholars. Compared with the fixed camera on the street, UAVs have stronger flexibility and can monitor and detect any range of public places, factories, and road traffic. In UAV aerial images, especially overhead images, crowd occlusion is rare. The main difficulty is that the human target is small, and too many down sampling times will lead to the loss of key information. Using mainstream target detection algorithms to detect and count pedestrians in UAV images is an effective method. The task locates the human body by learning the feature of the human body or head in the image information. The pedestrian counting result is the number of the located human bodies.

Early studies on pedestrian counting mainly focused on developing sliding windows to detect people, and then using this information to calculate the number of people [1]. For human detection, detection based on the whole human body or local detection is usually adopted. Detection based on the whole human body [2, 3] is a traditional pedestrian detection method. These methods train the classifier through the feature information extracted from the whole. These features include the Haar feature [4], directional gradient histogram feature [5], and edgelet feature [6].

Since then, various machine learning algorithms such as support vector machine and the random Forest have improved the prediction results of varying degrees, but these methods will be affected by high-density people and have limitations. To solve this problem, researchers used local detection [7–9] by estimating the number of people in the specified area by constructing a classifier based on face, head, or shoulder. With the development of convolutional neural networks and target detection technology, more and more deep neural network models are applied to counting tasks. For example, [10] completes the counting of people through face detection, and [11] uses the YOLO algorithm to complete the counting of people through human body detection. Literature [12] proposed a new network structure SAF R-CNN to train special subnetworks for large and small target pedestrians and capture their unique characteristics.

The counting method based on regression is generally used in the crowd counting scene. By learning the characteristic information corresponding to the crowd in the image, the number of people can be regressed directly, or we can regress to the crowd density map, and then calculate the number of people from the crowd density map. At present, in the field of population counting based on regression, the deep convolutional neural network has become a research hotspot and is widely used in many scenes. Cong et al. first proposed the crowd counting model Crowd CNN based on neural network in 2015 [13]. The model has a six-layer convolutional neural network, which realized the most effective performance on UCSD and other data sets at that time. Wang et al. proposed a seven-layer convolution neural network model and achieved good results on the UCF data set [14]. Zhang et al. proposed a multi-column convolutional neural network structure to map the image into a population density map and proposed a labeled ShanghaiTech data set [15]. Liu et al. proposed an end-to-end trainable deep network structure, which can use the features obtained by multiple receptive fields of different sizes, learn the importance of each feature in the image position, adaptively encode the scale of context information, and put forward the prospect of counting on the UAV platform [16]. Jiang et al. proposed a method to reduce the counting error caused by the difference of population density. The method has two networks, namely DANet (Density Attention Network) and ASNet (Attention Scale Network). DANet provides ASNet with attention masks related to regions of different density levels. ASNet first generates density maps and scaling factors, and then multiplies them by attention masks to output separate attention-based density maps [17]. The following problems still exist in the above research progress:

1. Small targets in UAV images are easy to be ignored, and key information is easy to be lost after multiple down sampling.
2. The background of aerial images is complex, it is difficult to pay more attention to the target area, especially in scenes with a sparse number of pedestrians. The method of generating density map is easy andleads to large errors in the number of people counted.

We proposed an end-to-end small target pedestrian detection and counting network model named YOLO-CC (YOLO and Crowd counting) based on UAV aerial images. The following are the main novelties and contributions of this study:

1. CSPDarknet-34 is used as the backbone network for feature extraction and the down sampling times of the original YOLOv4 are adjusted.
2. The density map generation network module is embedded into our model, which can generate the density map, calculate the number of people, and enhance the attention of the backbone to the target area.
3. The multiscale convolutional neural network is applied to the density map generation module.

The results show that the proposed method has a good performance on small target pedestrian detection and counting, and has a strong real-time performance. On the VisDrone2021-counting data set, the AP_50_ value is 39.32% and the MAE is 7.29.

## 2. Related Work

YOLO [18] is the first proposed one-stage target detection algorithm based on deep learning. The algorithm learns and extracts the features of the whole image through neural networks to predict each boundary box and the categories of all objects in the image at the same time. Firstly, the input image is divided into an *S* × *S* grid. Each grid cell needs to predict *B* bounding boxes and *C* categories. Each bounding box contains 5 parameters: *x*, *y*, *w*, *h* and confidence. The confidence is expressed as the IoU (Intersection over Union) between the prediction box and the real box. Finally, an s tensor is outputted, and the above information is mapped to this tensor. After decoding the information, the NMS (nonmaximum suppression) method is used to remove the duplication. The anchoring technology is introduced in YOLOv2 [19], which uses the offset between the anchor and the real frame to locate the target, normalizes the output of each layer, and accelerates the convergence speed of the network. The output images are feature maps with the size 13 × 13, which are obtained by five down sampling from images with the size 416 × 416, and these feature maps can meet the detection of most targets, but cannot meet the needs of multiscale target detection. YOLOv3 [20] uses Darknet-53 as the backbone network, which increases the depth of the network, and extracts three feature layers for result prediction. The scale of the feature map is 13 × 13, 26 × 26, and 52 × 52, respectively. Feature maps of different scales are used to predict targets of large, medium, and small size in images, respectively. YOLOv4 adopts the FPN (feature pyramid network) model for feature fusion in the output feature map, which improves the detection accuracy of small targets.

**Figure 1:**
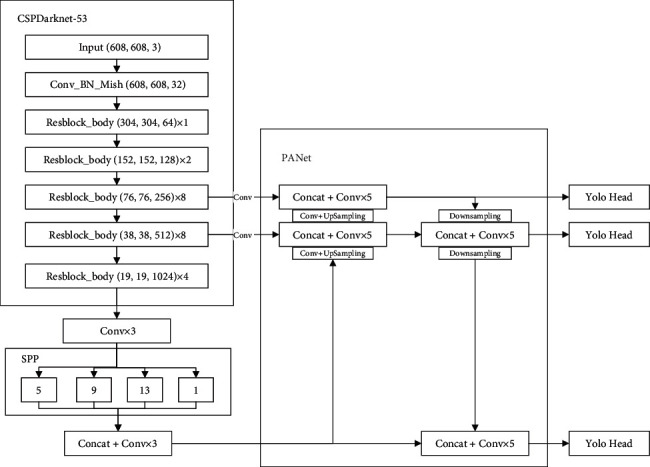
YOLOv4 structure.

YOLOv4 [21] adopts the CSPDarknet-53 as the backbone network for feature extraction, which further increased the detection accuracy while keeping the network depth unchanged. The neck of YOLOv4 uses the PANet (Path Aggregation Network) for feature fusion. SPP (spatial pyramid pooling) is used to expand the receptive field, which uses the maximum pooling method of *k* = [1 × 1, 5 × 5, 9 × 9, 13 × 13], and then concatenates the feature maps of different scales. The main differences of the YOLO series are given in Table 1.  Figure 1 shows the YOLOv4 structure.

## 3. Proposed Work

**Figure 2:**
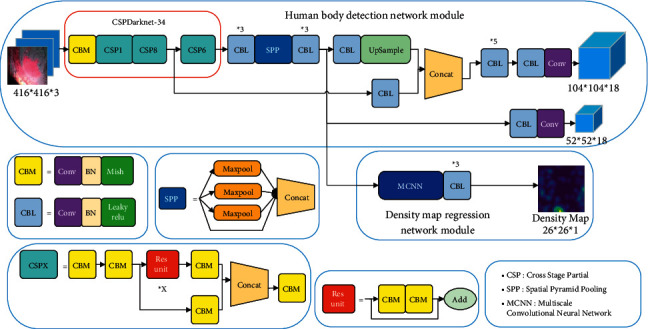
YOLO-CC structure.

The YOLO-CC network model we designed consists of the human body detection network module and the density map regression network module. The overall structure of the network is shown in Figure 2. The main goal of the human body detection network module is to locate and mark the human body. The main goal of the density map regression network module is to generate a density map to estimate the number of pedestrians and strengthen the attention to the target area in the process of feature extraction.

### 3.1. Human Body Detection Network Module

Due to too many parameters and too many down sampling times of the original feature extraction network model CSPDarknet-53, it easily cause the problems of slow convergence and network degradation. We optimized the network structure with lightweight-constructed CSPDarknet-34 as the backbone feature extraction network and reduced the number of convolution layers in the residual network from 53 to 34. The size of input image scale is 416 × 416 and the number of channels is 3. The main function of the first convolution unit is to increase the number of channels to 32, and the subsequent convolution operation is mainly composed of three CSP (Cross Stage Partial) residual units. The number of residual blocks contained in each CSP residual unit is 1, 8, and 6, respectively. Each residual block consists of a trunk composed of two convolution layers and a residual edge. Each convolution block in CSP residual unit is composed of a convolutional layer, a batch normalization layer, and a mish activation function. The purpose of the batch normalization layer is to make the distribution of network parameters of each layer as consistent as possible and accelerate the convergence speed. Compared with the ReLU activation function, the mish activation function is smoother at zero point and has stronger generalization ability. Mathematically, the mish activation function can be described as follows:

(1) $$\begin{array}{c} f\left(x\right)=x*\;\tan\;h\left(\text{ln}\left(1+\exp\;x\right)\right), \end{array}$$

where *x* represents the input matrix.

CSPDarknet-34 includes three down sampling operations. Each down sampling operation is completed by a convolution layer with a step of 2 and a convolutional kernel size of 3 × 3. The extracted effective feature images are C2 and C3, with dimensions of 128 and 256 and scales of 104 × 104 and 52 × 52, respectively. After the C3 feature map is output, it is sent to SPP to extract spatial feature information under different sizes. Then, after three convolutional layers, the dimension of the C3 feature map is reduced to 128, the main purpose is to summarize the effective features and reduce the amount of subsequent calculations. FPN is used for feature fusion. Its purpose is to combine the position information of the low-level feature layer with the semantic information of the high-level feature layer. The specific method is to splice the C3 with the C2 after sampling, output the YOLO head, and then the C3 outputs the YOLO head after a few operations. The target location is based on the anchor, because the target size of the data set is small, we only use two scales of anchor, 10 × 10 and 15 × 15, respectively. The max-IOU matching algorithm is used to count the matching degree between the ground truth and anchor, and select the largest matching anchor as the prediction box of the current target.

### 3.2. Density Map Regression Network Module

**Figure 3:**
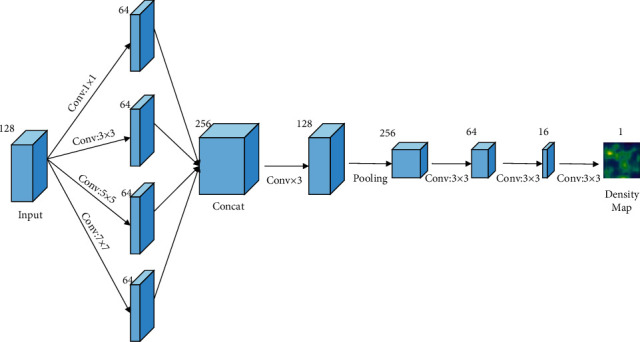
Density map regression network module structure.

In order to make the network model more intuitively output the crowd density in the image and make the feature extraction pay more attention to the target area, inspired by the way of generating mask graph in [22] to enhance the attention to the target area in the process of target detection and training, we designed the density map regression network module to generate crowd density graph. Its structure is shown in Figure 3. As the input of the MCNN (multiscale convolutional neural network) block, the C3 feature layer is convolved by four different convolutional kernels, and then concentrated together. The main purpose of this operation is to extract multiscale crowd image features. Images usually contain different sizes of head and aggregation information, so convolutional kernels with the same size are unlikely to capture the population density information at different scales. It is more natural to use convolutional kernels with different sizes to complete the mapping from original pixels to density maps. The size of convolutional kernels are 1 × 1, 3 × 3, 5 × 5, and 7 × 7. The number of channels of the characteristic graph after concentration is 256, and it is reduced to 128 after three convolution operations. Then, the feature map is pooled through the convolution operation with a convolutional kernel whose size is 3 and step size is 2, and then the size of the pooled feature map changes to 26 × 26. Finally, the convolutional layer with three convolutional kernels is used to reduce the number of channels in the feature map until the number of channels is 1, which is the final population density map. The parameter settings of each layer of the density map generation module are shown in Table 2.

We use a simple but intuitive way to generate the crowd density map. If there is a head at the position of *x*_*i*_ in the image, the corresponding position is expressed as  *δ*(*x* − *x*_*i*_), and the image with *N* people can be expressed as

(2) $$\begin{array}{c} H\left(x\right)=\sum_{i=1}^{N}\delta \left(x-x_{i}\right). \end{array}$$

**Figure 4:**
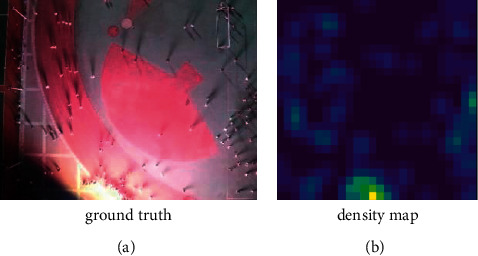
The actual effect of the density map generated. (a) Ground truth. (b) Density map.

Then, the image is transformed into a density map by Gaussian kernel function:

(3) $$\begin{array}{c} F\left(x\right)=H\left(x\right)\cdot G_{\sigma }\left(x\right), \end{array}$$

where *G*_*σ*_(*x*) is the Gaussian kernel function. Specifically, we first adjust the original image to 26 × 26, add 1 to the pixel point with head, and the pixel value of other areas without a head is 0. The total number of people is the sum of image pixel values. Then, Gaussian filtering with a kernel size of 3 × 3 is used to process the image in the form of density map, which can avoid the final output of the model converging to all 0, and the total population count remains unchanged. The actual effect of the density map generated by this method is shown in Figure 4. The number of people in the image can be calculated by summing the values of all pixels in the density map.

## 4. Experiments and Evaluation

We implemented the proposed YOLO-CC on Pytorch, the models are trained and tested with NVIDIA GeForce RTX 3090. Our CUDA vision is 11.4, the CPU model is Intel I9–10900K. In the human body detection module, the coordinate error adopts the mean square error, and the errors of classification and confidence adopts the cross entropy loss function. In the density map regression module, the error between the predicted density map and the real density map adopts the MAE (mean absolute error), and the final error is the sum of the above errors. During training, the size of input image is uniformly adjusted to 416 × 416.

Learning rate with cosine annealing function, the highest learning rate in the first 30 epochs is 1 × 10^−3^, followed by a maximum learning rate of 1 × 10^−4^ with a minimum learning rate of 1 × 10^−6^.

### 4.1. Data Set and Evaluations Metrics

The data set we used is VisDrone2021-counting [26], from the 2021 Vision Meets Drones: A Challenge. The data set is divided into two parts: train- and test-challenge, including 1807 and 912 RGB images, respectively. Test-challenge is dedicated to the testing in contests and does not provide real labels. Therefore, the images used in this paper are from train and are divided into training sets, a verification set and a test set in the ratio of 7 : 1 : 2. For the evaluation of pedestrian detection quality, we adopt metrics AP50 (average precision). Specifically, for AP50, to consider a bounding box prediction as true, the IoU between the predicated and the ground truth box must be higher than 0.5. For the evaluation of counting quality, we adopt MAE (mean absolute error) and MSE (mean squared error).

### 4.2. Experimental Results

**Figure 5:**
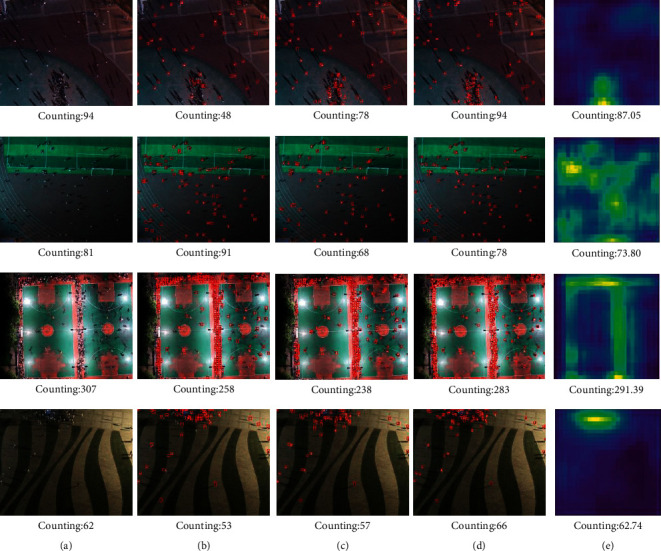
The visualization results of YOLOv4, YOLOX, and YOLO-CC in test set. (a) Ground truth. (b) YOLOv4. (c) YOLOX. (d) YOLO-CC human body. (e) YOLO-CC.

In this section, we evaluated two modules of YOLO-CC on the test set and compared with other methods. We used the original YOLOv4 as the baseline.  Table 3 shows the comparisons with other existing methods and reports all experimental results. Our model achieves the best results in AP_50_, MAE, MSE, and other evaluation metrics. Compared with the baseline, our model improves the AP_50_ metrics by 21.76%, MAE reduced from 11.36 to 7.29, and MSE reduced from 19.90 to 12.38. YOLOX is also a common method of object detection and achieved 5.12% improvement in AP_50_, our method is 16.64% higher than YOLOX in AP_50_. Visualization results of YOLO-CC, YOLOv4, and YOLOX in the test set is shown in Figure 5. YOLO-CC has a good performance in the test set. The results of human body detection generated by YOLO-CC can better give the specific location of the human body. The density map can more accurately reflect the actual population distribution.

### 4.3. Ablation Study

To validate the contributions of density map regression network module and MCNN block to the improvement of detection performance, respectively, we carry out ablation experiments on test set with YOLO-CC. As shown in Table 4, we gradually add modules to our model, the first row shows the performance of the baseline. From the second to the last row, AP_50_ gradually increased to 39.32 from 37.29, and MAE/MSE gradually decreased to 7.29/12.30 from 10.46/17.40. After adding the density map generation module, the AP_50_ metrics increases by 2.03%, which can effectively improve the accuracy of detection and counting. The main reason is that the module can improve the attention of the backbone network to small targets in the training process. After moving out of the MCNN block, the index decreases, because the MCNN block can learn to fuse the features of multiple scales.

### 4.4. *K*-Fold Validation Experiment

**Figure 6:**
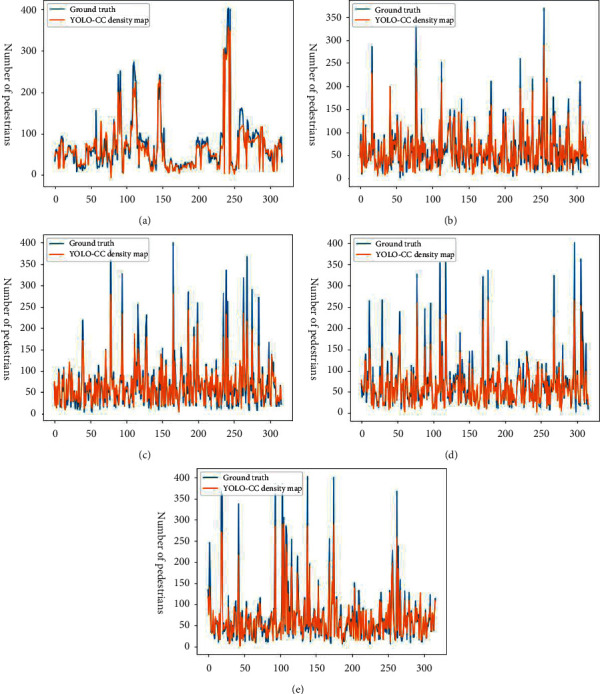
The comparison of the estimated number and the actual number of pedestrians in the density map generated by the YOLO-CC model.

The *K*-fold validation experiment means dividing the data set into *K* parts equally, choosing one of them as a test set to evaluate the model performance and training other *K* − 1 parts as a training set to train the model parameters, and then evaluating the model performance comprehensively based on the results of multiple groups. In our experiment, *K* takes 5.  Figure 6 shows the comparison of the estimated number of pedestrians and the actual number of pedestrians in the density map generated by the YOLO-CC model.  Table 5 shows the result of K-fold validation experiment. The experimental results show that the YOLO-CC model performs smoothly on different test sets. The estimated number of pedestrians in the density map fits the actual number of pedestrians, with an average error of 12.19.

## 5. Conclusion

This paper designs the YOLO-CC network model, which is divided into the human body detection network module and the density map regression network module. In the human detection network module, first of all, CSPDarknet-34 is used as the backbone network for feature extraction, after that SPP and FPN are used for feature enhancement and fusion, and finally fixed scale anchor is used for location and detection. In the density map regression network module, first of all, multiscale convolution is used to extract the features, and then the feature dimension reduction method is used to generate the predicted density map. Our experiments show that the human body detection network module can get better pedestrian detection results, and the density map regression network module can improve the attention to the target area and give better feedback about the pedestrian distribution.

In future, we will focus on more complex aerial images, such as dense crowds, to improve the accuracy of population detection and counting.

## Figures and Tables

**Table 1:** The main difference of YOLO series.

Model	Backbone	Feature map	Feature fusion	Number of anchors
YOLO	Conv × 24	7 × 7	-	0
YOLOv2	Darknet-19	13 × 13	-	5
YOLOv3	Darknet-53	13 × 13/26 × 26/52 × 52	FPN	9
YOLOv4	CSPDarknet-53	13 × 13/26 × 26/52 × 52	PANet	9

**Table 2:** The parameter settings of each layer of density map generation module.

Type	Input	Kernel size	Output
MCNN	52 × 52 × 128	(1/3/5/7) × (1/3/5/7)	52 × 52 × 256
Conv	52 × 52 × 256	(1/3/1) × (1/3/1)	52 × 52 × 128
Downsampling	52 × 52 × 128	3 × 3	26 × 26 × 256
Conv	26 × 26 × 256	3 × 3	26 × 26 × 64
Conv	26 × 26 × 64	3 × 3	26 × 26 × 16
Conv	26 × 26 × 16	3 × 3	26 × 26 × 1

**Table 3:** Comparisons with other existing methods in test set.

Models	Backbone	AP_50_	MAE	MSE	FPS
YOLO v4 (baseline)	CSPDarknet-53	17.56	11.36	19.90	26
CenterNet [23]	ResNet50	17.67	13.84	30.38	22
YOLOX [24]	Darknet-53	22.68	14.13	28.64	14
RFBNet [25]	VGG	17.99	9.58	19.27	32
YOLO-CC (Human body detection)	CSPDarknet-34	**39.32**	**7.29**	**12.38**	**34**
YOLO-CC (density map)		—	11.41	17.25	

**Table 4:** The ablation study of Density map regression module and MCNN in test set.

Method	Density map regression module	MCNN block	AP_50_	MAE	MSE
YOLO-CC	—	—	37.29	10.46	17.40
	√	—	37.75	8.86	15.03
	√	√	**39.32**	**7.29**	**12.38**

**Table 5:** The result of K-fold validation experiment.

Test set	AP_50_	MAE of density map
A	39.32	11.68
B	43.23	10.62
C	39.37	13.35
D	39.74	13.46
E	41.17	11.87

## Data Availability

All data included in this study can be downloaded from the official websites of “VisDrone–Vision Meets Drones: A Challenge” or can be obtained by contacting the corresponding authors.
